# SenseCare: a research platform for medical image informatics and interactive 3D visualization

**DOI:** 10.3389/fradi.2024.1460889

**Published:** 2024-11-21

**Authors:** Guotai Wang, Qi Duan, Tian Shen, Shaoting Zhang

**Affiliations:** ^1^ School of Mechanical and Electrical Engineering, University of Electronic Science and Technology of China, Chengdu, China; ^2^ SenseTime Research, Shanghai, China

**Keywords:** artificial intelligence, medical image informatics, clinical research platform, data annotation, model training

## Abstract

**Introduction:**

Clinical research on smart health has an increasing demand for intelligent and clinic-oriented medical image computing algorithms and platforms that support various applications. However, existing research platforms for medical image informatics have limited support for Artificial Intelligence (AI) algorithms and clinical applications.

**Methods:**

To this end, we have developed SenseCare research platform, which is designed to facilitate translational research on intelligent diagnosis and treatment planning in various clinical scenarios. It has several appealing functions and features such as advanced 3D visualization, concurrent and efficient web-based access, fast data synchronization and high data security, multi-center deployment, support for collaborative research, etc.

**Results and discussion:**

SenseCare provides a range of AI toolkits for different tasks, including image segmentation, registration, lesion and landmark detection from various image modalities ranging from radiology to pathology. It also facilitates the data annotation and model training processes, which makes it easier for clinical researchers to develop and deploy customized AI models. In addition, it is clinic-oriented and supports various clinical applications such as diagnosis and surgical planning for lung cancer, liver tumor, coronary artery disease, etc. By simplifying AI-based medical image analysis, SenseCare has a potential to promote clinical research in a wide range of disease diagnosis and treatment applications.

## 1 Introduction

With the development of medical imaging techniques and computer science, computer-aided systems for medical image analysis and downstream diagnosis and treatment decision have been playing an increasing role in clinic practices. In recent years, Artificial Intelligence (AI) has lead to a revolution of image analysis and pattern recognition, and has a huge potential to be applied for more efficient and intelligent medical image computing in a wide range of medical departments towards smart healthcare. However, before AI is used in clinic practice, extensive research studies are needed through the collaboration between clinicians, radiologists, pathologists, surgeons, AI scientists and engineers, which can validate the effectiveness, robustness, reliability and security of AI systems. To this end, a research platform that supports different medical image processing tasks and intelligent medical image computing algorithms for various clinic applications are highly desirable.

Despite the availability of several existing platforms for developing AI algorithms for general image recognition or medical image computing, they are not clinic-oriented and have limited support for clinical research. For example, TensorFlow (1), Pytorch (2) and Keras (3) are general deep learning libraries that provide low-level functions to develop complex deep learning models without specific functionality for medical image computing. Some other libraries such as NiftyNet (4), DLTK (5) and PyMIC (6) are developed for deep learning with medical images, but they are mainly designed for AI algorithm developers and there is no graphic user interface, which is difficult for clinicians and radiologists to use in specific clinic applications.

On the other hand, several medial image analysis solutions have also been developed in the past decades. Tools that solve a specific medical image processing task such as segmentation (7), registration (8, 9) and visualization (10) can be used for a part of a clinic application pipeline, but still not ready-to-use for clinic researchers. Some research platforms such as MITK (11), NifTK (12) and 3D Slicer (13) provide several traditional medical image analysis tools and 3D visualization for image guided intervention. However, these platforms have limited support for AI models. Recently, some AI-based plugins have been added to 3D Slicer for image segmentation tasks, but the AI models for other tasks are limited, and it does not support model training and dealing with pathological images. Recently, some virtual reality visualization platforms such as COVI3D (14, 15) have been proposed for quantification and interpretation of lung tumor and lesions (16, 17) with the help of AI models, but their extensibility to other applications is limited. In the era of multi-modal medical data, a desired clinical research platform based on AI does not only need to provide AI models for various clinical applications, but also require data exchange with other image management systems, functionality across diverse image modalities, availability of sophisticated 3D visualization and high extensibility and portability for different clinic scenarios.

To solve these problems, we introduce the SenseCare Smart Health Platform (SenseCare for short) that aims to take advantages of state-of-the-art AI techniques to foster researchers from different clinical departments to implement innovations for improvement through the whole process of clinical diagnosis, treatment planning and rehabilitation management. Compared with some existing medical image computing and visualization platforms, it has the following functionalities and advantages: (1) Integrating a wide range of AI algorithms based on deep learning for extensive image processing tasks, including classification, detection, segmentation, registration and 3D visualization; (2) Supporting various data modalities ranging from structural data like radiological and pathological images to time series data like ECG, with real-time data synchronization among different systems; (3) Offering web-based access and multi-center deployment with high concurrency across different devices and operating systems; (4) Facilitating collaborative research for various clinical applications. SenseCare has increasingly assisted to achieve outputs in several research projects including quantitative analysis of cardiac function (18), assessment of knee articular cartilages (19), pathological image analysis (20, 21), lung cancer diagnosis (22, 23), quantitative brain tumor assessment (24, 25), spine image analysis (26, 27) and radiotherapy planning for head and neck cancers (28, 29), etc.

The following sections of this paper are organized as follows: In Section 2.1, we give a brief summary of the architecture of SenseCare, which is followed by detailed descriptions of basic functional modules in Section 2.2. We then introduce SenseCare’s AI toolkits in Section 2.3. In Section 3, we show several examples of clinical applications powered by SenseCare. Finally, discussions and conclusions are given in Sections 4, 5, respectively.

## 2 Material and methods

### 2.1 Architecture of sensecare

**Figure 1:**
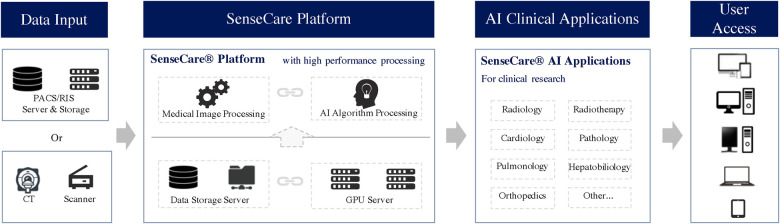
Architecture of SenseCare platform.

As shown in Figure 1, SenseCare provides a wide range of artificial intelligence algorithms based on deep learning for learning from and analyze different kinds of medical data. It also provides advanced visualization of medical images that enables users to analyze complex anatomies and segmented structures. These modules are combined with a browser/server architecture and multi-center deployment so that they are accessible on different kinds of devices and at various locations.

The architecture of SenseCare follows a modular structure that consists of three layers: (1) basic functional modules such as data management and visualization, web-based access and multi-center deployment, (2) advanced AI toolkits that include libraries for model training and many built-in AI algorithms for image segmentation, registration, lesion detection, etc., and (3) application scenarios that adapt the basic functional modules and AI algorithms to deal with different clinical tasks such as computer assisted diagnosis of the lung and surgical planning for bone tumors.

### 2.2 Basic functional modules

#### 2.2.1 Data support

**Support of various imaging modalities.** SenseCare supports intelligent analysis of in images in various modalities ranging from radiological images to pathological images. For radiological images, it allows efficient import, query, retrieval, and storage of clinical images using DICOM protocols and structures. Major radiological images including Computed Tomography (CT), Magnetic Resonance Imaging (MRI), Digital Radiology (DR) and Positron Emission Tomography (PET) are all well supported by SenseCare in different clinical applications. For pathological images, SenseCare supports several image formats including SVS, TIFF, VMS, NPDI, KFB and others. It also provides a series of functions of import, query, retrieval, storage, management, common measurements and analysis to help pathologists perform diagnosis in a more efficient and intuitive way.

**Data synchronization and security.** Medical data are commonly stored in different systems such as the Picture Archiving and Communication System (PACS) and Radiology Information System (RIS). Synchronization of data between these systems and the image computing workstation is critical in a clinical research environment. To facilitate more efficient and functional workflow for clinical researchers and medical practitioners, SenseCare can be seamlessly integrated into existing information systems in the hospital, and provides users with improved access by efficient data synchronization. It is capable of synchronizing data from PACS, RIS and other common information systems in hospitals without disturbing the original clinical workflows. When newly acquired data are transmitted or changes in status take place in these information systems, SenseCare will synchronize the updated information and present the users with the latest information automatically. It also supports pulling data directly from PACS/RIS based on user-defined rules. For example, users can designate image modality and type or time range, and send queries to fetch the data they want from a database.

Several strategies are employed by SenseCare to ensure data security in clinical environment. First, the sever of SenceCare is located in the hospital, and is only accessible in the local area network within the hospital, avoiding the risk of leaking data to the internet. Secondly, the cloud-based image computing and visualization in SenseCare avoids data transmission from the server to users’ devices, and only the outputs of image analysis and rendering are sent to the client side. Thirdly, encryption algorithms are embedded in SenseCare to reliably manage and protect user accounts and data from potential risk factors.

#### 2.2.2 Advanced visualization

**Figure 3:**
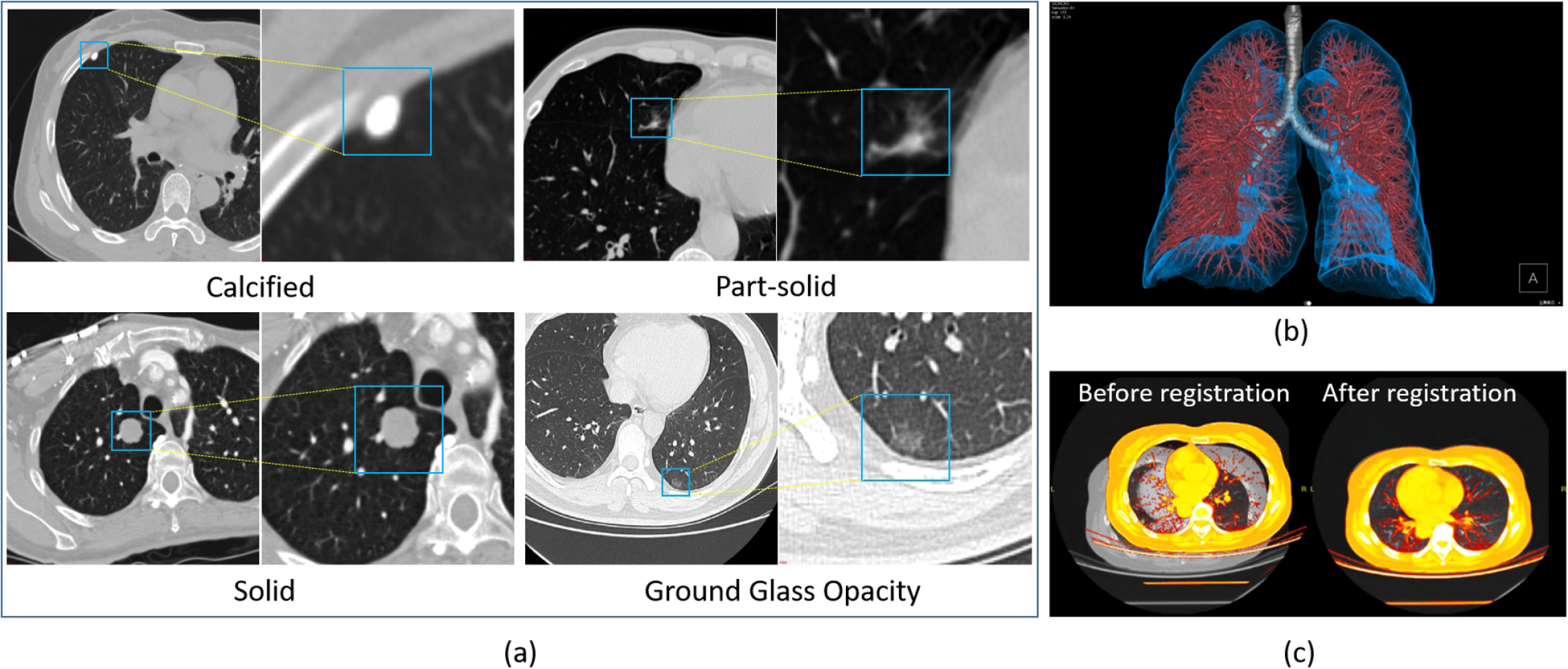
Nodule detection, image registration and 3D visualization of lung images in SenseCare. **(a)** Lung nodule detection. **(b)** 3D visualization. **(c)** Image registration.

**Figure 4:**
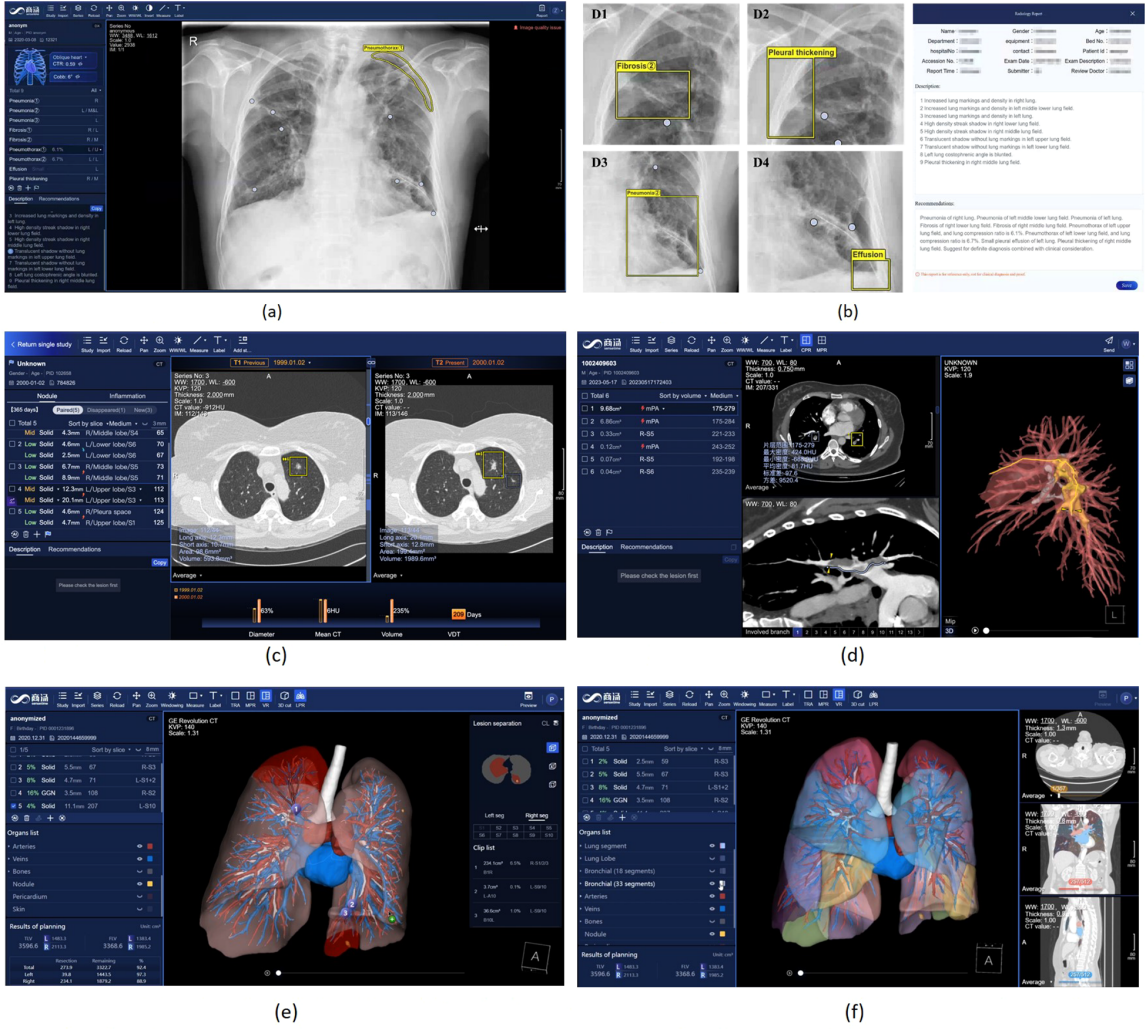
Chest X-Ray image and Lung CT analysis in SenseCare. **(a)** User interface of Chest X-ray analysis. **(b)** Multi-disease detection and automated report generation. **(c)** Intelligent lung nodule analysis and follow-up in CT. **(d)** Intelligent analysis of pulmonary embolism. **(e)** Lung surgical planning with visualized traced tube structures. **(f)** Lung surgical planning with lung segment analysis.

SenseCare provides advanced 3D reconstruction and visualization of medical images to facilitate the analysis of complex anatomies and segmented structures, which presents data and information in a more explicit way thus improving the information interpretation. Comprehensive methods such as Maximum Intensity Projection (MIP), Minimum Intensity Projection (MinIP), Multi-Planar Reconstruction (MPR), Curved Planar Reformation (CPR) and 3D volume rendering are available for users to perform 3D visualization and enhance interactivity. These capabilities play an important role in clinical diagnosis meanwhile contributing to surgical planning, simulation and navigation, as well as radiotherapy planning, etc, as shown in Figures 3c, 4.

#### 2.2.3 Concurrent and efficient web-based access

SenseCare is designed with high performance concurrency. The network communication structure of SenseCare enables hundreds of users to concurrently perform high-resolution image rendering and 3D post-progressing thus satisfying multiple usage needs. With servers deployed, SenseCare platform allows over 1,000 concurrent users to review and retrieve medical images while its comprehensive toolkits for advanced image post-processing are designed to support more than 160 users simultaneously.

SenseCare can be used in different devices and operating systems and no plugins are needed. By adopting a browser/server architecture, it offers a truly seamless user experience and eliminates the need for multiple logons. Users can access not only MPR, MIP/MinIP, CPR tools, but also the full range of three-dimensional capabilities through HTML5 websites. SenseCare grants radiologists and physicians with efficient access and workflow-boosting benefits even when working from iPad, smartphone or laptop.

#### 2.2.4 Multi-center deployment

Collecting data from multiple centers is important for developing robust algorithms and large-scale clinic studies. To support such research that requires a collaboration between researchers from different locations, SenseCare can be deployed at multiple centers. This favorable feature distinguishes SenseCare from traditional image computing workstations that are located at a single institution.

In addition, SenseCare’s multi-center deployment facilitates the data collection and access process. Various types of data from different centers can be collected and cleaned under standardized rules. Data scientists, clinical researchers, and pharmacists are all able to participate in the process while respecting the original clinical process.

#### 2.2.5 Support for collaborative research

To facilitate the collaboration between researchers, SenseCare provides a specific Document Management System (DMS) to make it easier to organize, secure, capture, digitize, tag, approve and complete tasks with research-related files. It can handle a large amount of papers and images and cope with different workflows by supporting advanced user permission management and task management. This efficient tool enables researchers to manage multiple sophisticated research projects easily, and helps a ground of researchers collaborate with each other and exchange knowledge for efficient accomplishment of comprehensive research projects.

For user permission management, SenseCare supports different levels of users. Admin users can create normal users and set permissions for other normal users to access, view, manipulate, share and remove folders and files. Nonetheless, Admin users can change the permission if needed. For task management, SenseCare’s DMS allows admin users to break down tasks and delegate tasks to normal users. It also allows users to track task progress from the beginning to the end and set small milestones to make sure the whole project will be finished on time. Users can prioritize, organize and set deadlines for themselves and are able to draw together the resources they need to achieve their research goals.

### 2.3 Artificial intelligence toolkits

Recent years have seen a fast growing of novel deep learning algorithms for medical image computing tasks (30), which play an important role for more accurate and efficient diagnosis and treatment planning and assessment. In this section, we introduce SenseCare’s AI toolkits for medical image computing, including tools for users to develop new algorithms, and built-in deep learning models for image segmentation, registration and lesion detection, etc.

#### 2.3.1 Data annotation and model training

Deep learning models require a large amount of annotated data for training, and both annotation of medical images and model training are time-consuming and complex for common clinical researchers. To facilitate developing AI models for a range of medical image analysis tasks, SenseCare not only provides AI-assisted image annotation tools to improve annotation efficiency, but also offers a framework to train deep learning models easily, and support docker integration to avoid complex environment configuration by users.

**Efficient data annotation tools**. To facilitate the annotation of a large set of training images for developing AI models, SenseCare provides a set of off-the-shelf tools for efficient image annotation, such as contouring tumors and organs for segmentation tasks, bounding box annotation for object and lesion detection tasks. The annotation tools in SenseCare are available for both radiological images and pathological images, and they provide a variety of annotation types. For example, users can choose from different interactive styles including mouse click points, rectangles, circles, ellipses, polygons or hand-drawn shapes based on the characteristics of the target.

**Figure 2:**
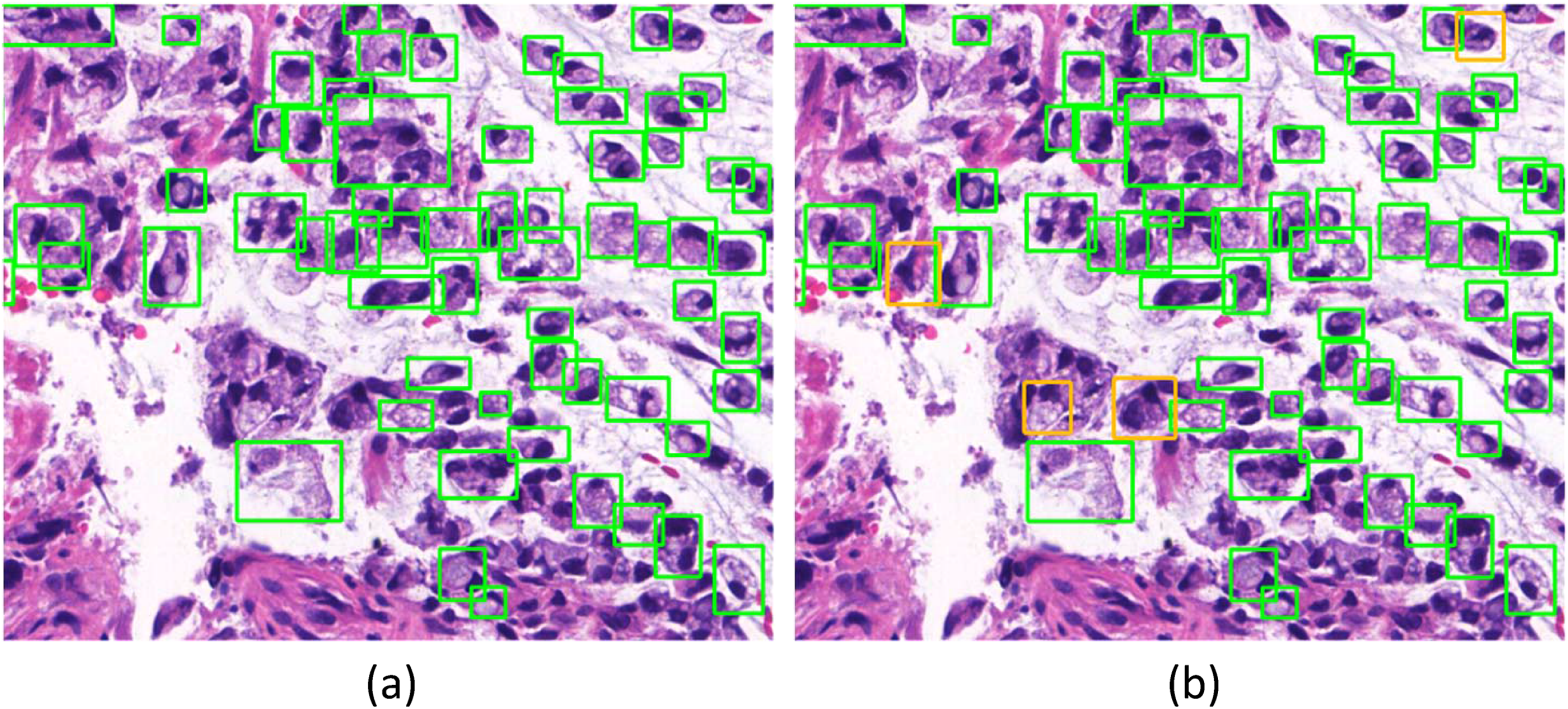
Annotation of signet ring cell carcinoma. **(a)** Suggested annotations. **(b)** Manual refinement.

Since manual annotation is time-consuming and annotation may vary from different annotators’ inputs, SenseCare also supports semi-automatic annotation with minimal user interactions and high efficiency and accuracy. For medical image segmentation, the interactive annotation tool not only generates high-accuracy boundaries based on the click/bounding box prompt given by the user (31), but also automatically suggests the most informative samples for annotation (32), which largely reduces the annotation burdens and improves the efficiency. For object detection tasks, the annotator can start from annotations automatically generated by algorithms, and only needs to provide few interactions to obtain refined annotations. As an example, Figure 2 shows efficient annotation of signet ring cell carcinoma in pathological images using SenseCare, where algorithms suggested some annotations in Figure 2a, and the annotator only provides manual refinement to obtain accurate annotations for a slide in Figure 2b.

**Model training**. SenseCare’s AI toolkits are constructed on the basis of SenseParrots, a deep learning framework independently developed by SenseTime. While matching mainstream frameworks such as TensorFlow (1) and PyTorch (2), it also has advantages in ultra-deep networks and ultra-large-scale data model training. The integrated optimization from the underlying layer to the SenseParrots framework enables the platform to outperform others under the same level of configuration. By using more than 1,000 GPU cards for parallel training, it takes less than 1.5 min to complete the AlexNet neural network training in a benchmark task, surpassing the previous fastest 4 min record (33).

For medical image analysis, SenseCare supports a wide range of neural network structures including CNN-based and ViT-based networks for image classification, detection and segmentation, etc. Considering that the there are a large amount of unannotated images or imperfect annotations in many applications, SenseCare also provides Semi-Supervised Learning (SSL) and Weakly Supervised Learning (WSL) methods for training. Especially, SSL leverages a small set of annotated images with a large set of unannotated images for training, and can better mine information from unannotated cases based on pseudo labels or consistency regularization (34). WSL leverages image-level or sparse annotations for training high-performance models (35), which reduces the annotation requirement. Additionally, noise-robust loss functions and training paradigms are implemented in SenseCare to learn from noisy labels (36), which prevents the model being corrupted by incorrect or noisy labels in the training set.

SenseCare provides a human-in-the-loop strategy for data annotation and model training. For a set of unannotated images, it first allows the user to label some images using the interactive annotation tools. With a set of labeled images (and optionally a set of unlabeled images for SSL), it then invokes the model training process through a configuration file, where configurations include the paths of training data and output model, training strategies (e.g., fully supervised learning or SSL), network structure, loss function, optimizer and hyper-parameters such as batch size, learning rate and training epoch number. Additionally, users can select image pre-processing and augmentation methods in the configuration file. After training, the model can be used to predict labels for remaining unlabeled images with uncertainty estimation, which is used to query the user for active learning-based annotation. The training process is iteratively conducted with more annotations obtained. The human-in-the-loop strategy avoids labeling all the images from scratch and improves the annotation efficiency. In each round of training, SenceCare allows the user to use a set of validation dataset to validate the performance and assess the degree of over-fitting, and the training can be early stopped if the performance on validation set does not increase for several epochs. Note that a single round of training is also allowed if the user has provided annotations for the training images in advance.

**Docker integration**. Users of SenseCare can employ the built-in deep learning models mentioned above for several image computing tasks, and they can also develop their own models with the help of SenseCare’s training and testing pipelines. When the user creates a new algorithm, SenseCare provides a dockerized version of the algorithm, and can package it with all the dependencies together in the form of containers, so that the algorithm can work seamlessly in any new environment. Therefore, researchers can focus on the development of AI algorithms without worrying about the testing and production environment. Since dockers are lightweight, SenseCare makes it more convenient and efficient for users to develop, test and deploy algorithms and deep learning models for various clinical applications.

#### 2.3.2 Object detection

Automatic detection of objects of interest is a common task for computer assisted diagnosis systems. SenseCare has several built-in deep learning models for object detection tasks, such as landmark and lesion detection in 3D radiological images and cancer cell detection in histopathological images.

For example, automatic localization of vertebrae in CT is important for image-guided diagnosis, pre-operative planning and post-operative evaluation. Deep learning models such as 3D Fully Convolutional Neural Networks (FCN) are embedded in SenseCare for accurate vertebrae localization (26). The model also takes advantages of prior knowledge such as spatial and sequential constraints to obtain high robustness in challenging cases. In addition, SenseCare is able to predict tumor invasiveness and malignant in Ground Glass Opacity (GGO) on the basis of its lung nodule detection model (22). Figure 3a shows an example of lung nodule detection by deep learning models in SenseCare. For signet ring cell carcinoma detection from Hematoxylin and Eosin (H&E) stained Whole Slide Images (WSI), SenceCare is integrated with bottom-up approaches (37) that obtain cell instance masks first and then derive bounding boxes for each instance, which is more accurate than the general RCNN-based detection methods.

#### 2.3.3 Image segmentation

Image segmentation is essential for most clinical applications such as accurate modeling of anatomical structures, quantitative measurement of tumor volumes, planning of radiotherapy and surgical treatment. Its output has a large impact on the downstream workflows. However, due to the low contrast between the target tissue and its surroundings, inhomogeneous appearance, complex shape variation and image noise, accurate segmentation is extremely challenging and traditional image segmentation algorithms are often faced with large regions of over- and under-segmentation.

Supported by its deep learning-based image segmentation models, SenseCare can overcome these challenges and has obtained state-of-the-art performance in a range of segmentation tasks. For example, to segment the complex structures of pulmonary vessels from CT images, SenseCare is equipped with a multi-view-based 2.5D network with a low complexity, which outperformed other contemporary networks by a large margin on the LIDC dataset (38). To segment intervertebral discs from MR images, a novel multi-resolution path network with deep supervision is included in SenseCare and it achieved superior performance on the MICCAI 2018 IVDM3Seg challenge dataset (27). Several specific CNN models are also developed for other applications, such as multiple Organs-at-Risk (OAR) segmentation from CT for radiotherapy planning (28), segmentation of optic disc and cup for glaucoma diagnosis (39), nuclei instance segmentation in histopathological images (20, 21) and cartilage segmentation from MR images for osteoarthritis assessment (19). These different models are ready-to-use and serve as strong baselines for the above specific applications, and they can be easily adapted to new segmentation tasks based on the user’s research interests.

#### 2.3.4 Image registration

Images acquired in different modalities are often need to be fused to provide sufficient information for diagnosis and treatment decision. In addition, a patient may be scanned several times at different stages of a disease to obtain a better understanding of the evolution of the disease. Therefore, it is necessary to register two or more images into a common spatial coordinate system for better interpretation of anatomical and pathological characteristics thus improving diagnosis and treatment for patients.

SenseCare provides several registration algorithms for different tasks. For example, the combination of MR and CT is quite useful since the former is better suited for delineation of tumor regions while the latter is needed for accurate computation of the radiation dose. Registration between MR and CT images supported by SenseCare makes it more efficient and accurate to obtain radiotherapy planning. Registration algorithms embedded in SenseCare include both rigid and non-rigid registration between images from a single modality or multiple modalities. Rigid methods are useful for the registration in the presence of rigid bodies such as bones. Non-rigid registration is used for applications such as correcting soft-tissue deformation during imaging and modeling the dynamic heart. Figure 3c shows an example of non-rigid lung image registration in SenseCare.

#### 2.3.5 Foundation models

Recently, foundation models such as vision-language models (40) and Segment Anything Model (SAM) (41) have shown great potential in reducing data and annotation cost and improving performance in applications with limited dataset size (42). SenseCare currently supports using foundation models mainly in two ways for researchers. First, it allows researchers to use foundation models to efficiently annotate data in image classification and segmentation tasks. For example, by leveraging the zero-shot inference ability of vision-language models such as variants of CLIP (40), users can first obtain pseudo-labels of training images. Then, SenseCare uses uncertainty estimation to select reliable pseudo-labels, and leverages semi-supervised learning methods to learn from those with reliable pseudo-labels and other unlabeled samples. SenseCare also supports source-free domain adaptation of medical image segmentation models with the help of SAM (43). Specifically, pseudo-labels obtained by the source model is used to generate prompts for SAM that outputs improved pseudo-labels for target-domain images. The new pseudo-labels are then used to fine-tune the source model with prior knowledge-based constraints (44), which makes the source model be well adapted to a new domain without annotations.

Second, SenseCare provides a foundation model that improves the performance of downstream tasks and reduces the annotation cost (45). A hybrid architecture combining the advantage of CNN and Transformers is designed, and it is trained on a large-scale CT dataset based on a volume fusion-based self-supervised learning framework. Then the pretrained model can be adapted to different downstream segmentation targets including head and neck organs, thoracic/abdominal organs and lesions in CT and other modalities.

## 3 Clinical applications

The most important high-level goal of SenseCare is to serve as a platform for clinical research in various applications. In this section, we give several examples of user scenarios where SenseCare is adapted for various clinical applications.

### 3.1 Lung-oriented application

#### 3.1.1 Chest radiograph multi-disease detection

Chest Radiography (CR) serves as the most common imaging tool for lung disease screening and diagnosis. SenseCare offers a comprehensive CR-based multi-disease detection system, encompassing foreign body detection, screening for negative findings, multi-disease detection, rib counting, cardiothoracic ratio measurement, scoliosis calculation, and automated report generation, as shown in Figures 4a,b. Specifically, SenseCare begins by performing quality control on the input chest radiograph. It then detects 14 diseases, including Abnormal Aortic Knob, Atelectasis, Cardiomegaly, Edema, Emphysema, Fibrosis, Hilar Enlargement, Nodule, Pleural Effusion, Pleural Thickening, Pneumonia, Pneumothorax, Rib Fracture, and Tuberculosis, providing bounding box and confidence score for each disease. For pneumothorax, it further calculates the pneumothorax compression ratio by segmenting the pneumothorax and lung regions using UNet (46). Rib instance segmentation and detangling are implemented to achieve rib counting and determine the corresponding regions for disease boxes. The cardiothoracic ratio is calculated by detecting key points that define the chest contour and heart width. Cobb angle measurement for scoliosis is achieved through spine segmentation and extraction of the spinal centerline (47). Through the coordination of multiple modules, SenseCare offers an automated, rapid, and comprehensive fine-grained diagnosis for CR.

#### 3.1.2 Lung nodule analysis and follow-up

Lung cancer is a malignant disease with a five-year survival rate of 16%–17%. Researches have shown that early diagnosis and intervention will improve the five-year survival rate up to 54%. Although computer-assisted image analysis have been adopted to facilitate the lung cancer detection and diagnosis in an early stage, the various sizes, shapes and types of pulmonary nodules are placing great burdens on clinical physicians, thus causing misdiagnosis and missed diagnosis due to their fatigue and overwork.

Based on its leading algorithms, SenseCare supports a thorough research and analysis of pulmonary nodules and lesions (36) by automatic detection, segmentation, and quantitative analysis. It can automatically detect and locate the nodules (22, 48) and then provide further quantitative information of each nodule such as its volume and density, in addition to qualitative estimation of its type and malignancy. As shown in Figure 3, four kinds of nodules are automatically detected and distinguished by SenseCare (23). Furthermore, it equips clinicians with an advanced nodule follow-up feature. Utilizing registration technology, it facilitates the longitudinal analysis between any two patient scans, automatically pinpointing and correlating the positions of existing, vanished, or emergent nodules. It meticulously gauges alterations in nodule size, density, and malignancy risk, estimating the lesion’s doubling time, which is a crucial metric that informs clinical decision-making. Figure 4c illustrates the user interface for thoracic CT imaging analysis and smart follow-up within SenseCare. Compared with COVI3D (14, 15) that is specifically designed for lung lesion analysis, SenseCare provides a more detailed analysis and visualization of other important thoracic structures such as different lung lobes, vessels and airways.

#### 3.1.3 Pulmonary embolism analysis

Pulmonary Embolism (PE) is formed when a portion of a blood clot breaks off from the wall of a vein and travels through the blood stream, passes through the heart (right atrium and right ventricle), and becomes lodged in a pulmonary artery, causing a partial or complete obstruction. The best available diagnostic technique of PE is Computed Tomography Pulmonary Angiography (CTPA) (49), where PEs usually appear as dark spots among the bright regions of blood arteries.

Leveraging CTPA data, SenseCare employs advanced algorithms for detection, segmentation, and localization, enabling the automated identification, positioning, and quantitative analysis of pulmonary embolisms (50). The system proactively issues high-risk alerts when blockages are present in the principal pulmonary artery. Furthermore, it performs curved plane reconstruction for each individual embolus, streamlining the examination of the embolism’s overall state. The curved plane reconstruction technology vividly illustrates the embolus’s position and morphology within the vessel, enhancing physicians’ capacity to evaluate the condition. In addition, SenseCare provides intelligent 3D reconstruction for clinicians. By offering a 3D visualization of various anatomical structures such as the lungs, pulmonary arteries and veins, pericardium, and embolism, it eases the assessment of the proximity of emboli to neighboring tissues, furnishing a crucial benchmark for subsequent thrombolytic treatments. Figure 4b illustrates the user interface for CTPA imaging PE analysis within SenseCare.

Table 1 shows the performance of SenseCare and related works on lung CT analysis. It obtains a sensitivity of 0.93 at 6 False Positives (FPs) per scan for lung nodule detection, and a Dice of 0.945 for lung lobe segmentation. It also supports pulmonary vessel and airway segmentation with an average Dice of 0.914, and the sensitivity for pulmonary embolism detection is 0.90 at 2 FPs per scan.

**Table 1:** Performance of SenseCare and related works on lung CT analysis.

Task	Works	Method	Training/Test cases	Performance
Lung nodule detection	Kuo et al. (51)	Support vector machine	381 in total	SEN = 0.92
	Zhu et al. (52)	3D Faster R-CNN	888 (10-fold CV)	SEN = 0.90@2FP/scan
	Ardimento et al. (53)	Ensemble networks	510/500	Recall = 0.9873, Precision = 0.8062
	SenseCare	Attention CNN	11,483/1,759	SEN = 0.93@6FP/scan
Lung lobe segmentation	Zheng et al. (54)	Dual-attention V-Net	40/10	Dice = 93.4%
	Xie et al. (55)	Cascaded CNNs	4,370/1,100	Dice = 93.6%
	SenseCare	3D Res-UNet	954/179	Dice = 94.5%
Pulmonary vessel & airway segmentation	Wu et al. (56)	Cascade U-Net	100/43	Dice = 71.7%
	Wu et al. (57)	3D UNet	42/14	Dice = 84.9%
	SenseCare	3D Res-UNet	308/132	Dice = 91.4%
Pulmonary embolism detection	Huang et al. (58)	Multi-phase CNN	6,292/1,000	AUROC = 92.58%
	Ma et al. (59)	Multi-phase CNN	1,428/369	AUROC = 85.47%, SEN = 0.75
	SenseCare	3D Res-UNet	2,069/130	SEN = 0.90@2FP/scan

CV, Cross validation; SEN, sensitivity; FP, False positives.

#### 3.1.4 Lung surgical planning

Surgical operations play an important role in effective treatment of lung cancer. Due to complex anatomical structures as well as breathing movements in chest, visualized surgical planning is essential for improving the efficiency and success rate of surgeries. SenseCare has provided the Lung Surgical Planning system that can automatically build 3D quantitative pulmonary structures from CT images, shown in Figures 4c,d. The whole pipeline of extracting various structures is completed in three minutes without manual interactions. Soft tissues including pulmonary parenchyma, pulmonary lobes and segments are extracted as analysis basis. Besides, the bronchi tree is traced and analyzed according to the position and depth, acting as important landmarks during resection. Furthermore, pulmonary arteries and veins are obtained following the vessel tree structures, which ensures the precise segmentation results even in non-contrast CT images. Base on above results, the system supports watershed analysis for precise lung resection, as well as precise needle placement suggestions for tumors and surrounding tissues in ablation treatment. The system helps clinicians obtain both global visualizations and important details, leading to more efficient and accurate operations in clinical practice.

### 3.2 Pathology-related application

Pathological diagnosis is regarded as the most reliable criteria for diagnosis of cancers such as Gastrointestinal cancer. However, it is labor-insensitive and time-consuming to manually discern lesion areas and pathological cells from up to 100,000 × 100,000-pixel whole slide pathological images, which easily leads to fatigue of human analysts and cannot satisfy the increasing demand for pathological diagnosis due to the lack of adept experts in developing countries.

**Figure 5:**
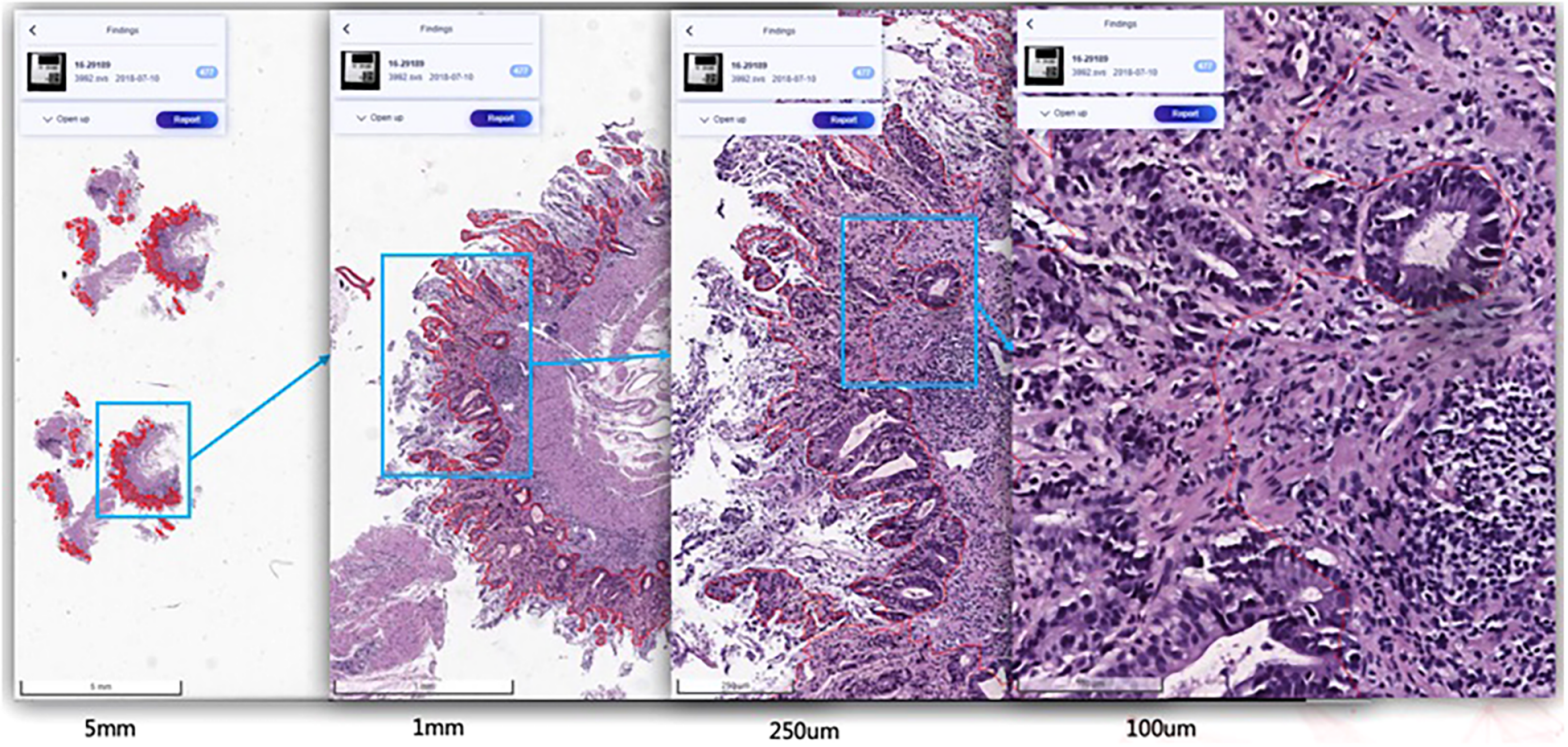
Pathological image analysis in SenseCare.

SenseCare-Pathology is designed to support pathological diagnosis across a spectrum of anatomical regions, including gastrointestinal, cervical, lymph nodes, lung, prostate and pleural effusion & ascites diseases, and it facilitates both tissue and cellular level analysis (37, 60). This innovative platform empowers pathology department of hospitals and third-party pathological diagnosis centers to perform efficient and intelligent analysis of pathological images by providing AI-based functional modules such as lesion and cell detection (37), nuclei/gland segmentation (21) and cell segmentation (61), etc. Especially, SenseCare supports efficient retrieval of pathological images from a large-scale database (61–63). Users can employ the retrieval module to search for similar images for a given input image. These modules help clinical researchers build capability to conduct large-scale cancer screening projects. Figure 5 shows an example of lesion localization from pathological images.

Table 2 shows the performance of SenseCare compared with some recent works on pathological image classification tasks. For gastric cancer malignancy detection from WSIs, it obtains an sensitivity of 0.95 and specificity of 0.91. For colorectal cancer malignancy detection, the sensitivity and specificity is 0.93 and 0.85, respectively. For Thin-prep Cytologic Test (TCT) analysis, the sensitivity and specificity of cervical cancer malignancy detection is 0.95 and 0.83, respectively. SenseCare also supports classification of mold from TCT (sensitivity 0.90 and specificity 0.99), classification of clue cells (sensitivity 0.92 and specificity 0.99), segmentation of cancer region in WSI (Dice 0.87) and detection of low-quality WSIs including the presence of bubble, glue, folding, contaminant with an overall sensitivity of 0.90.

**Table 2:** Performance of SenseCare and related works on pathological image analysis.

Task	Works	Training/test cases	Performance
Gastric cancer malignancy detection	Ba et al. (64)	2,123/110 WSIs	SEN = 0.906, SPE = 0.782
	Huang et al. (65)	2,333/175 WSIs	SEN = 0.934, SPE = 0.905
	SenseCare	20,000/3,000 WSIs	SEN = 0.952, SPE = 0.913
Colorectal cancer malignancy detection	Ho et al. (66)	105/150 WSIs	SEN = 0.97, SPE = 0.60
	Song et al. (67)	177/194 WSIs	SEN = 0.89, SPE = 0.79
	SenseCare	20,000/3,500 WSIs	SEN = 0.93, SPE = 0.85
Cervical cancer screening	Zhang et al. (68)	5,558/1,389 WSIs	SEN = 0.83, PRE = 0.88
	Cao et al. (69)	5,558/1,389 WSIs	SEN = 0.91, PRE = 0.89
	SenseCare	18,000/4,000 WSIs	SEN = 0.96, SPE = 0.83

SEN, sensitivity; SPE, Specificity; PRE, Precision.

### 3.3 Heart disease diagnosis

Cardiovascular diseases (CVDs) are the most common causes of death throughout the world. Non-invasive morphological and functional assessment of cardiovascular structures plays an important role in diagnosis and treatment of CVDs. Computed Tomography Angiography (CTA) and electrocardiogram (ECG) are two common means for diagnosis of CVDs. However, manual analysis of CTA and ECG is laborious and time-consuming. SenseCare provides a full-stack solution towards automatic diagnosis of coronary artery disease based CTA and ECG.

#### 3.3.1 Coronary artery disease diagnosis using CTA

For automatic diagnosis of coronary artery disease using CTA, SenseCare provides a full-stack solution, including fully automatic segmentation of 3D whole heart and coronary arteries, extraction of coronary artery centerlines, labeling of important artery branches, reconstruction of MPR and CPR, real-time volumetric rendering, detection of plaques and quantification of stenosis, and automatic generation of diagnose reports.

**Figure 6:**
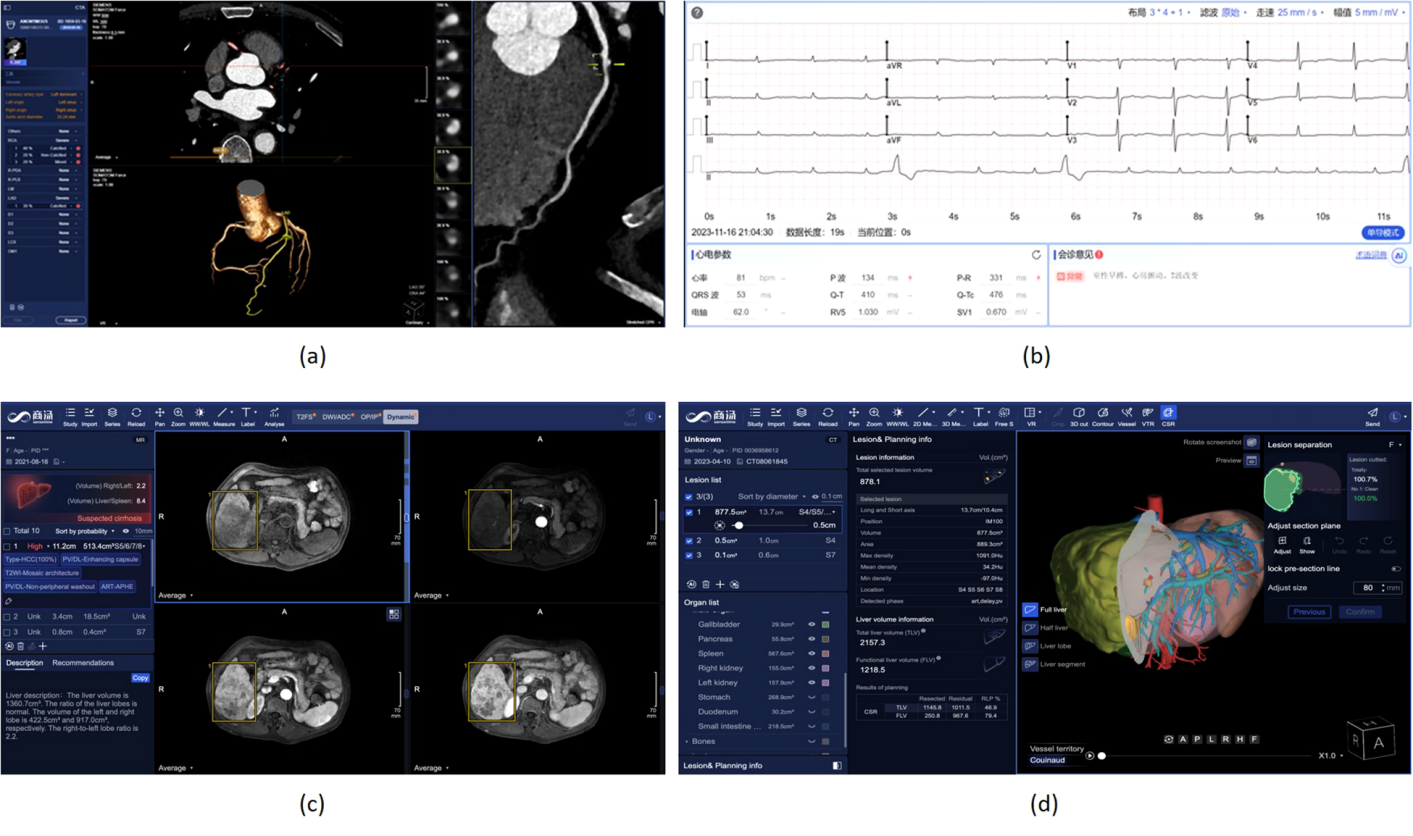
Heart disease and liver cancer diagnosis and surgical planning system in SenseCare. **(a)** Conroy artery disease diagnosis using CTA. **(b)** Cardiac disease diagnosis using ECG. **(c)** Liver cancer diagnosis system. **(d)** Liver surgical planning system.

More specifically, SenseCare employs a cascaded method for automatic segmentation of 3D whole heart (70) and coronary arteries, which is integrated into a semi-supervised framework (71) and has achieved state-of-the-art performance using very few manual annotations. For robust and accurate coronary artery segmentation, an Artery and Vein Disentanglement Network (AVDNet) is proposed by incorporating the coronary vein into the segmentation task (72). A unified deep reinforcement learning (73) framework is proposed to automatically traverse tree-structure centerlines of coronary arteries. Pixel-level segmentation followed by 3D classification and segmentation of point sets (74) is proposed for coronary artery labeling. We utilize a recurrent CNN to automatic detect and classify the type of coronary artery plaque (75), and the degree of coronary artery stenosis is quantified by a multi-class segmentation of plaque and vessel lumen from the reconstructed probe images. Finally, a structured diagnose report is summarized based on the results obtained from all steps mentioned above, and the whole procedure is finished within one minute. Figure 6a shows a snapshot of the coronary artery application in SenseCare.

Table 3 shows the performance of coronary artery and cerebral artery CTA analysis obtained by SenseCare compared with recent related works. For coronary artery analysis, SenseCare obtains a patient-level accuracy of 0.94 for stenosis detection (≥ 50% stenosis), with an accuracy of 0.97 for calcium score calculation and 0.96 for coronary artery labeling, respectively. For cerebral artery analysis, the accuracy of artery labeling is 0.95, and the sensitivity of intracranial aneurysm detection is 0.94. The sensitivity of cerebral hemorrhage detection is 0.99, with a specificity of 0.92. The sensitivity and specificity for cerebral ischemia detection is 0.88 and 0.95, respectively.

**Table 3:** Performance of SenseCare and related works on CTA analysis.

Task	Works	Training/test cases	Performance
Coronary artery stenosis detection	Zreik et al. (76)	126 (10-fold CV)	ACC = 0.71 for ≥50% stenosis
	Grifn et al. (77)	303 for evaluation	ACC = 0.84 for ≥50% stenosis
	SenseCare	1,600/400	ACC = 0.94 for ≥50% stenosis
Coronary artery calcium score	Mu et al. (78)	365/240	ACC = 0.93
	Hong et al. (79)	852/215	ACC = 0.95
	SenseCare	640/160	ACC = 0.97
Intracranial aneurysm detection	Dai et al. (80)	208/103	SEN = 0.92
	Hu et al. (81)	12,817/900	SEN = 0.94
	SenseCare	800/200	SEN = 0.94
Cerebral hemorrhage detection	Kim et al. (82)	180/20	SEN = 0.67, SPE = 0.86
	Wang et al. (83)	19,530/2,214	SEN = 0.95, SPE = 0.94
	SenseCare	640/160	SEN = 0.99, SPE = 0.92

CV, Cross validation; ACC, Accuracy, SEN, sensitivity; SPE, Specificity.

#### 3.3.2 Automatic diagnosis of cardiac diseases using ECG

As a common means of heart disease detection, ECG has the characteristics of being inexpensive, non-invasive, and easily available. The manual measurement of parameters and waveform recognition are essential steps in the traditional ECG diagnostic process, posing significant challenges for doctors when analyzing long signal sequences. As shown in Figure 6b, SenseCare provides an automatic solution for ECG diagnosis, including parameter measurement and diseases classification.

### 3.4 Liver cancer diagnosis and surgical planning

Primary liver cancer, mainly Hepatocellular Carcinoma (HCC), is the sixth most prevalent malignancy and third leading cause of cancer-related death worldwide. The prognosis is poor owing to the high recurrence in 60%–70% of patients within 5 years after curative surgery (84). Diagnosis of liver cancer based on multi-modal medical images is important for treatment decision.

SenseCare provides a closed-loop platform for intelligent diagnosis of liver cancer using multi-phase dynamic CT scans and multi-sequence MRI images. As shown in Figure 6c, it has an integrated pipeline for automatic sequence alignment, detection and localization of tumors (85). Moreover, it provides morphology and density measurements for assessment of cancer type, malignancy and LI-RADS symptom (86). It also implements automatic segmentation and volume measurements of abdomen organs, and risk assessment of cirrhosis and fatty liver. Finally, a structured and standardized diagnostic report is generated. Therefore, SenseCare serves as a promising tool to help radiologists locate and identify the suspected liver tumors more efficiently and effectively.

Table 4 shows the performance of SenseCare and recent relevant works on structure segmentation and lesion detection in liver CT images. For abdominal organ segmentation, the average Dice of liver, left kidney, right kidney and spleen from CT and MRI is 0.96 and 0.95, respectively. For hepatic vessel segmentation, the recall of center line is 0.88 in both CT and MRI. The sensitivity and specificity of lesion detection are 0.95 and 0.70 for CT images, and 0.92 and 0.70 for MRI images, respectively. For classification between benign and malignant liver lesions, the accuracy is 0.90, with an AUC of 0.95. SenseCare also supports automatic recognition dynamic phases of contrast enhanced CT with an accuracy of 0.97, and the classification accuracy for different MRI dynamic sequences is 0.97.

**Table 4:** Performance of SenseCare and related works on liver image analysis.

Task	Works	Training/test cases	Performance
Multi-organ segmentation	Shaker et al. (87)	18/12	Avg-Dice(CT) = 0.92
	Chen et al. (88)	18/12	Avg-Dice(CT) = 0.91
	SenseCare	670/66	Avg-Dice(CT) = 0.96, Avg-Dice(MRI) = 0.95
Liver lesion detection	Zhou et al. (89)	462/154	SEN = 0.93, Precision = 0.83
	Kim et al. (90)	761/589	SEN = 0.85@4.8FP/scan
	SenseCare	2,040/582	SEN = 0.95
Hepatic vessel segmentation	Xu et al. (91)	36/20	Dice = 0.69, SEN = 0.79
	Chen et al. (88)	443(5-fold CV)	Dice = 0.65
	SenseCare	372/52	Recall of center line = 0.88

CV, Cross validation; SEN, sensitivity; FP, False positives.

For liver surgical planning, SenseCare provides a quantitative 3D modeling of the liver for highly automated liver-specific analysis, which is shown in Figure 6d. It helps hepatobiliary surgeons perform interactive analysis and treatment planning. In detail, it supports efficient automatic segmentation and quantitation of tumors, vessels, ducts and abdomen organs within four minutes, and interactive refinement of segmentation results. It supports pre-surgical planning for liver resection in multiple paradigms including anatomical, vessel-territory-based and curvature-based methods. The system also aides in precise needle placement relative to tumors and surrounding tissues for ablation treatment. It helps clinicians get a whole picture of the abdomen in a quicker and more comprehensive paradigm, which improves the safety of operations and ensures the treatment outcome.

### 3.5 Pelvic tumor surgical planning

Pelvic tumor is one of highly malignant tumors, and surgical treatment is the most effective treatment for pelvis tumors, where an accurate preoperative simulation and planning based on segmentation and modeling of the tumor is critical for the success of surgery.

**Figure 7:**
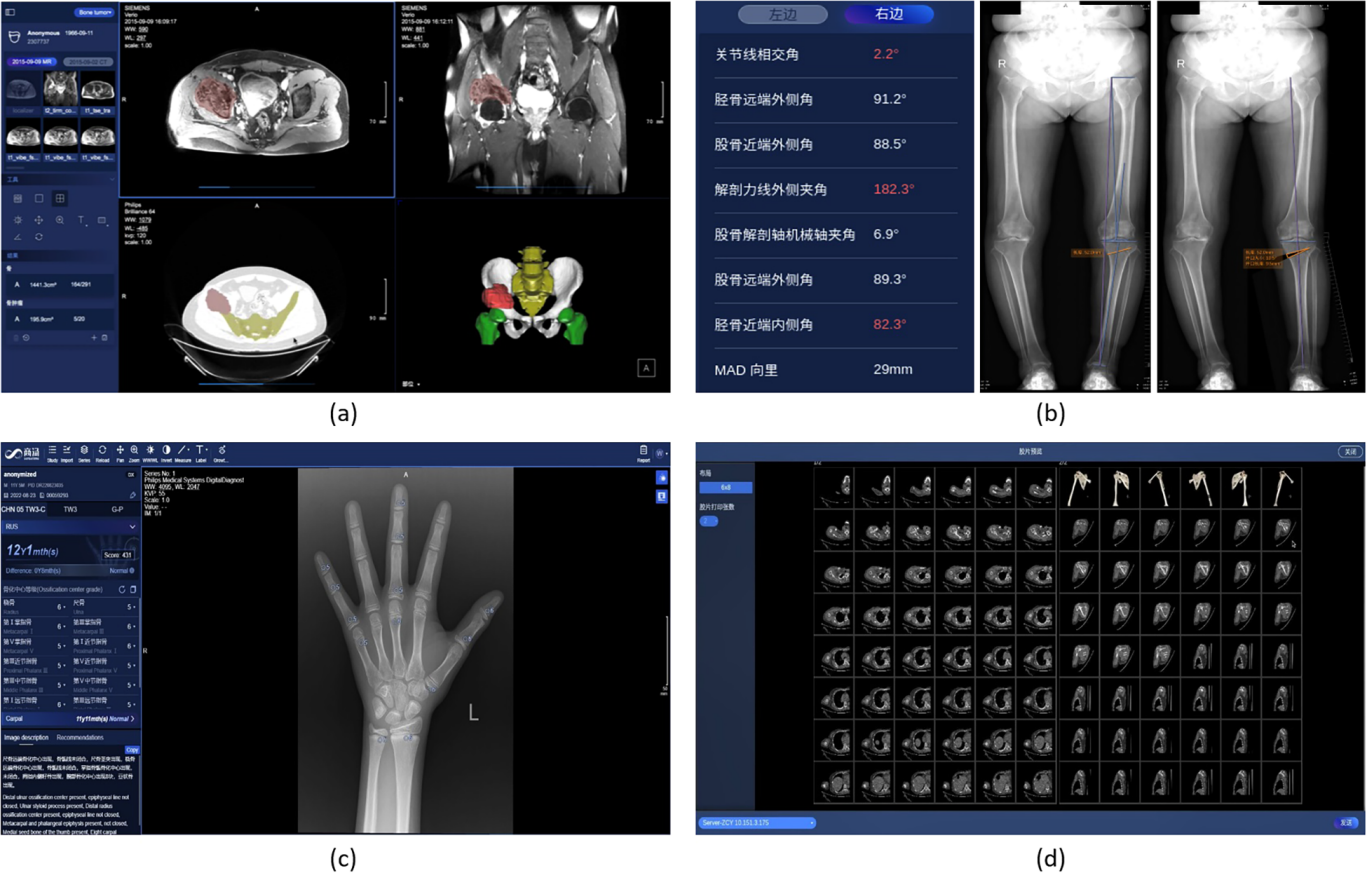
Pelvic tumor and skeleton-oriented applications in SenseCare. **(a)** Pelvic tumor surgical planning. **(b)** Lower limb surgical planning. **(c)** Bone age assessment. **(d)** Skeleton reconstruction.

SenseCare provides an intelligent preoperative surgical planning for for limb salvage surgery of malignant pelvic tumors, where the accuracy and efficiency is improved by our deep learning-based algorithms. The surgical planning workflow mainly consists of three parts. First, the pelvic tumor is segmented from MR scans with a U-Net like model (46). Then the pelvic bone is segmented from CT scans with a CNN combined with self-attention blocks (92). Finally, a robust rigid/affine inverse-consistency registration method that is an extension of SymMirorr (93) is conducted to align the corresponding MR-CT pair. Based on the registered CT-MR pair and the corresponding segmentation results, surgeons and radiologists could make accurate preoperative surgical planning rapidly. Figure 7a shows the user interface of pelvic tumor surgical planning system in SenseCare. The system efficiently cuts down the multi-party communication costs between radiology, orthopedics and 3D printing centers, and ultimately reduces doctor’s workload and facilitates patient-tailored treatment. It took only 15 min to complete the surgical planning for pelvic tumor resection, which is a dramatic acceleration compared with the 2-day time span in a traditional workflow (94).

### 3.6 Skeleton-oriented application

Skeleton-relevant diseases such as fracture, development disorder and osteoarthritis could largely reduce the patients’ quality of life, and lead to several complications. Skeleton analysis based on medical imaging is essential for accurate diagnosis and treatment planning of such diseases. SenceCare provides several intelligent tools for diagnosis and assessment of skeleton diseases, including lower limb surgical planning, bone age assessment and whole body skeleton reconstruction.

#### 3.6.1 Lower limb surgical planning

Knee Osteoarthritis (OA) is an important public health issue that causes chronic disability. Lower limb osteotomies is a well-established and commonly utilized technique in medial knee osteoarthritis secondary to different forms of knee joint malalignments, trying to establish a better alignment by passing the load bearing leg axis through hip, knee and ankle joint. The measurement of lower limb axial alignment with radiographs is a critical step in the preoperative planning to exactly define the characteristic of the osteotomy.

SenseCare’s lower limb surgical planning system provides advanced AI algorithms for automatic radiograph analysis such as segmentation of femur and tibia and detection of lower limb landmarks for accurate measurement of the critical angles and distances of lower limbs. The results are used for planning of surgical treatments such as High Tibial Osteotomy (HTO), as shown in Figure 7b. Localization of landmarks is based on a coarse-to-fine recurrent network (95) combined with Gaussian heatmap regression (96). The heatmap corresponding to a landmark location is a sum of multiple Gaussian functions centered at that landmark. At inference time, the largest values in the heatmap are taken as the detected landmark positions. Such a strategy helps to achieve stable and accurate limb landmark detection results that ensure the reliability of downstream surgical planning.

#### 3.6.2 Bone age assessment

Bone age is an important and widely used quantitative metric to estimate the development of child’s skeleton in the field of pediatrics. Greulich and Pyle (GP) and Tanner–Whitehouse (TW) are the two most used bone age assessment methods in clinical practice. The GP method requires comparing the radiograph with the reference atlases and taking the bone age of the closest one as the evaluation result. Yet TW method is much more complex due to its scoring mechanism. The radiologist needs to manually evaluate the developmental stages of 20 specific bones. Each developmental stage of each bone corresponds to a score. By adding the scores of all bones, the final bone maturity score is obtained, and the bone age is calculated. Obviously, this is a time-consuming and error-prone task, and a fully automated workflow is desirable.

SenseCare provides a fast and fully automated solution that integrates multiple bone age assessment methods, including TW3 RUS, TW3 Carpal, and GP. As shown in Figure 7c, it can automatically detect 20 specific bones in the hand X-ray image, evaluate the development status of each bone, and calculate the bone age based on different methods. Meanwhile, an assessment report containing descriptions of primary developmental characteristics is also presented.

#### 3.6.3 Whole body skeleton reconstruction

The SenseCare intelligent Virtual Reality (VR) application is oriented to the whole body’s skeleton and joints, and fully automatically performs VR and standard plane reconstruction to help users interpret scans. For the VR function, the target skeleton are fully automatically distinguished from the background including non-interesting bones and plaster fixation, thereby eliminating irrelevant information during VR display. For standard plane reconstruction, the shape and posture of the target joint (including shoulder, elbow, wrist, knee, ankle, foot, etc.) in the image can be automatically recognized, and then the scan is reconstructed along a preset standard direction, as shown in Figure 7d.

### 3.7 Stroke diagnosis

Stroke is a severe cerebrovascular disease globally, with high incidence, high disability rate and mortality (97). Ischemic stroke is the most common type of stroke, accounting for 75%–85% of all stroke cases. The detection and quantitative evaluation of stroke lesions by medical imaging is of great significance for accurate diagnosis and treatment decision.

**Figure 8:**
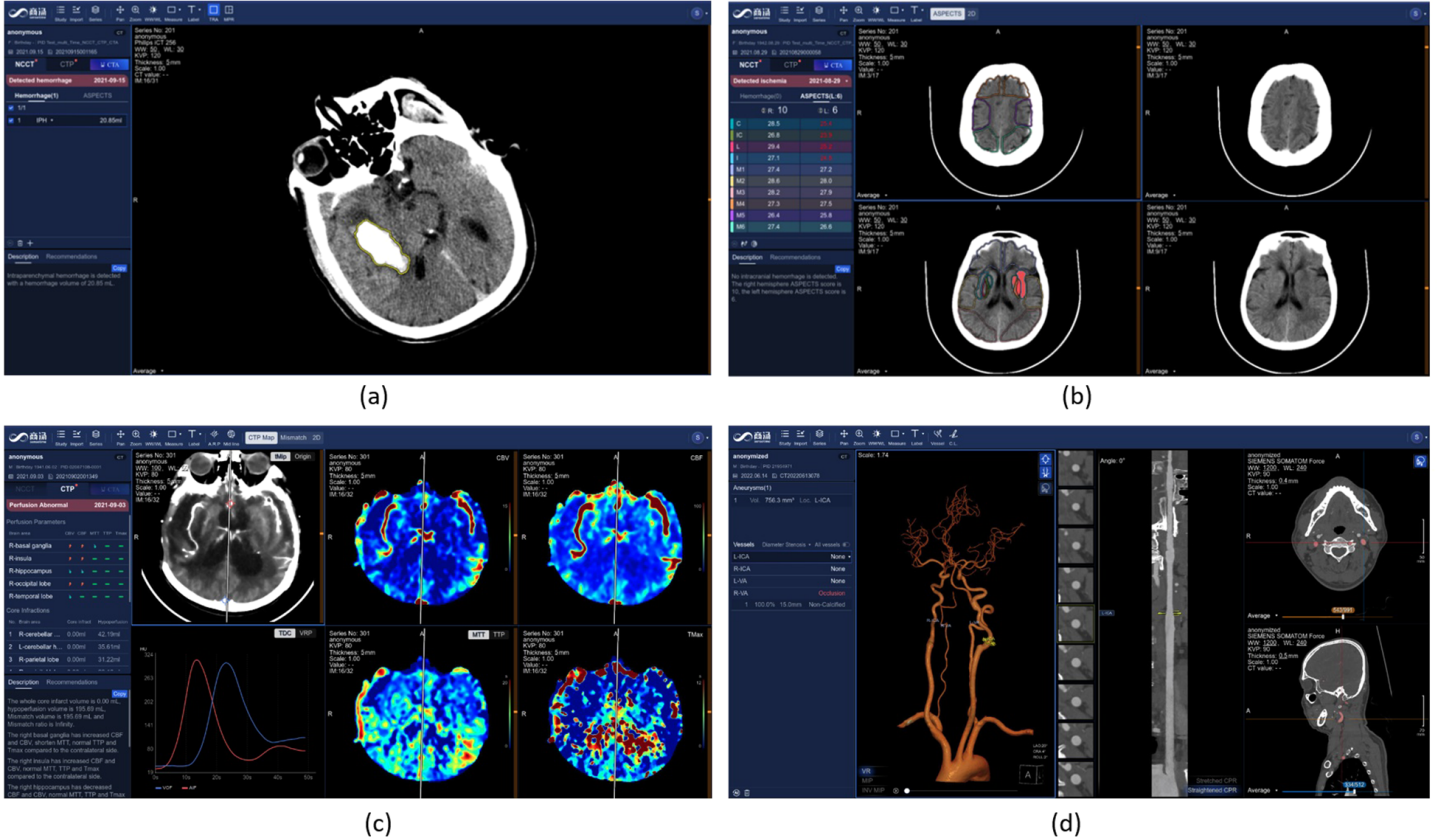
Stroke diagnosis in SenseCare. **(a)** Hemorrhage diagnosis. **(b)** ASPECT score measurement for ischemic stroke. **(c)** CT perfusion analysis. **(d)** Head and neck CTA analysis.

The SenseCare platform provides a one-stop stroke diagnosis solution based on different types of CT imaging. First, it provides automatic analysis of intracerebral hemorrhage and ischemic stroke using non-contrast CT. As shown in Figure 8a, for intracerebral hemorrhage, it can detect the hemorrhage and calculate the volume of bleeding automatically, and classify the hemorrhage into five types. For ischemic stroke, the ASPECT score is automatically measured for preliminary assessment of the severity of ischemia, as shown in Figure 8b. Second, for ischemic stroke, the CT perfusion analysis system can decode raw 4D CT perfusion images into perfusion parameter maps (CBV, CB, MTT and TMAX) for accurate diagnosis, as shown in Figure 8c. It can quantify the volume (98) and mismatch between the infarct core and the ischemic penumbra. Thirdly, for thrombectomy patients, SenseCare features a head and neck CTA reconstruction and analysis system (99) for rapid vascular reconstruction and localization of plaque occluded areas and aneurysm, as shown in Figure 8d. In terms of quantitative performance, the sensitivity and specificity for infarcted region detection is 0.92 and 0.94, respectively, and the relative volume error for low-perfusion region segmentation is 5.0%. The holistic solution and high performance in CT perfusion image analysis can be applied to stroke centers to improve their treatment capacity.

### 3.8 Radiotherapy contouring

Radiotherapy is an important and widely-adopted treatment for cancers. In radiotherapy treatment planning, one of the most critical step is the delineation of the organs-at-risk (OAR) and target volumes, the quality of which is a core factor affecting the efficacy and side effects of radiotherapy. Clinically, radiologists have to spend several hours for manual delineation, due to the large number of OARs, complex shapes of cancers and large size of 3D volumes. As a result, it requires great time and efforts, as well as a high level of professionalism for the radiologist.

**Figure 9:**
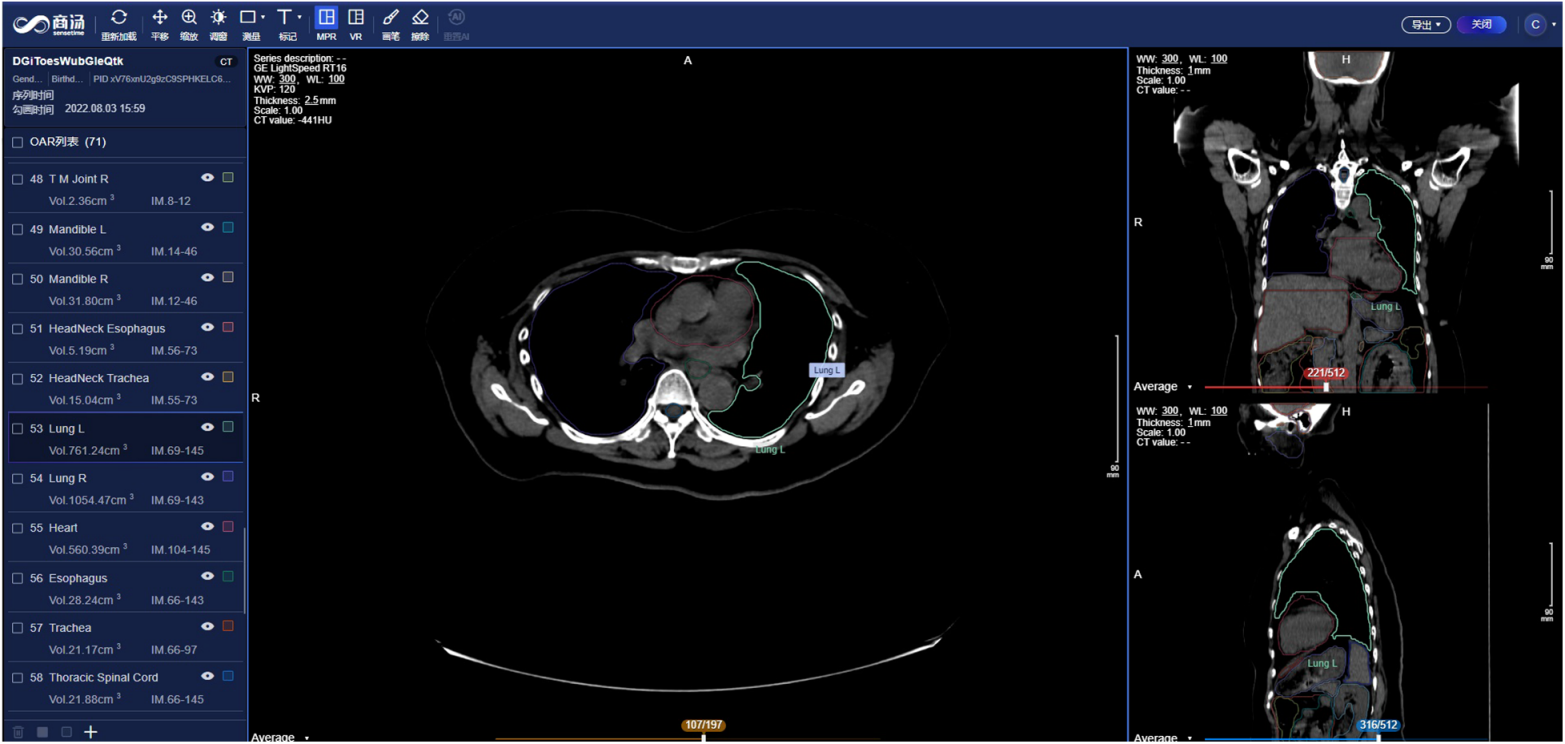
Radiotherapy contouring system in SenseCare.

As shown in Figure 9, SenseCare Radiotherapy Contouring system aids in the radiologists by automatically contouring OARs across the whole body, including the head and neck (100–103), chest (104) and abdomen (105, 106), as well as common targets including breast cancer, rectal cancer (107), etc. The system can be seamlessly connected to different kinds of CT machines and Treatment Planning Systems (TPS), automatically run AI calculation in background, support a 3D interactive view and editing the delineated structures, and export the results back via the standard RT structure DICOM protocol, in a complete closed loop of workflow.

## 4 Discussion

With the development of medical imaging techniques and artificial intelligence, AI-based medical image computing systems have an increasing potential for more intelligent diagnosis and treatment in clinic practice. However, existing deep learning platforms rarely support specific clinical applications while most current medical image analysis platforms are not well supported by advanced AI algorithms. A desirable research platform for AI-based medical image informatics should have easy-to-use and extensive AI modules, be accessible to clinical researchers at multiple centers and on various devices, and support a range of clinical applications with different data modalities. To achieve this goal and boost clinical research towards smart healthcare, SenseCare is developed as a generic research platform for intelligent medical image computing that can support various research needs across different medical disciplines.

To analyze how SenseCare meets the above goals, we collected some feedback from users. 53 clinical users from 9 hospitals at 4 different cities were asked to assess to which extent they agreed or disagreed (a 7-point scale) with 8 statements: (Q1) SenseCare helps a lot in image analysis for diagnosis; (Q2) SenseCare is easy to learn and operate; (Q3) SenseCare helps to improve the diagnosis performance of users; (Q4) It effectively reduces the risk of missed diagnosis for lesions especially small lung nodules and rib fracture; (Q5) SenseCare has a good performance for 3D vessel reconstruction for coronary artery CTA, head and neck CTA and brain Magnetic Resonance Angiography (MRA); (Q6) SenseCare’s 3D reconstruction of anatomical structures based on segmentation is helpful for surgical planning in lung and liver cancers; (Q7) SenseCare improves the efficiency and performance in follow-up of patients; (Q8) Expect to use more AI tools in SenseCare to assist solving problems in healthcare. The summary of agreement level is shown in Table 5. 51 out of 53 users (96.23%) agreed or strongly agreed that SenseCare is helpful for diagnosis (Q1), and 52 out of 53 users agreed or strongly agreed that SenseCare is easy to learn and use (Q2). More than 46 (86.79%) users agreed or strongly agreed that SenseCare helps to improve the capability of users (Q3), reduces missed diagnosis (Q4), obtains good results in vessel reconstruction (Q5) and leads to better follow-up (Q7). In terms of being helpful for surgical planing, 51 users gave the first three agreement levels, accounting for 96.23%. In average, 90.81% users agreed or strongly agreed with the 8 statements.

**Table 5:** Agreement level of 53 clinical users on 8 statements of SenseCare.

Level	Q1	Q2	Q3	Q4	Q5	Q6	Q7	Q8	Average
Agree (strongly)	38	37	34	35	32	28	32	43	65.81%
Agree	13	15	13	12	14	14	15	10	25.00%
Agree (somewhat)	2	1	3	5	6	9	5	0	7.31%
Neutral	0	0	2	0	0	2	0	0	0.94%
Disagree (somewhat)	0	0	0	1	1	0	0	0	0.47%
Disagree	0	0	1	0	0	0	1	0	0.47%
Disagree (strongly)	0	0	0	0	0	0	0	0	0.00%

As shown in Table 6, compared with existing platforms of medical image analysis and visualization for clinical applications, SenseCare has several advantages: (1) On-the-shelf AI toolkits for different medical image computing tasks such as image segmentation, registration, lesion and landmark detection, which makes it easy for users to not only directly use these built-in AI models for specific tasks, but also develop new customized AI models based on SenseCare’s tools for intelligent image annotation, model training and validation; (2) Extensive clinical applications that are developed for a range of clinical diagnosis and treatment planning tasks, and the comprehensive AI functional modules and advanced 3D visualization make SenseCare scalable for new clinical applications; (3) Easy to access with high concurrency and low requirement on users’ device or operating system, due to its cloud-based service and multi-center deployment; and (4) Efficient synchronization of data from different information systems and cross multiple centers, with data privacy ensured by encryption algorithms.

**Table 6:** Comparison between SeneseCare and existing medical image computing and visualization platforms for clinical applications.

Platform	Deep learning algorithms	Visualization	Model training	Pathology	Multiple applications	Web-based access
MITK Nolden et al. (11)	×	✓	×	×	✓	×
3D Slicer Pieper et al. (13)	✓	✓	×	×	✓	✓
COVI3D Benbelkacem et al. (14)	✓	✓	×	×	×	×
SenseCare	✓	✓	✓	✓	✓	✓

Despite that a range of AI toolkit have been available in SenseCare for many clinical applications, there are still some aspects that need to be improved. First, the current foundation models in SenseCare are mainly for general radiology and pathology image analysis, and foundation model for other modalities such as surgical videos will be considered in the future (108). Second, SenseCare currently focuses mainly on structured medical data. However, many healthcare scenarios require the integration of unstructured data such as clinical notes and patient histories. We will expand its support for unstructured data to enhance the platform’s ability to provide comprehensive diagnostic insights in the future. Thirdly, to better support precision medicine, prediction of prognosis, treatment responses and survival rates of patients will be supported by SenseCare, which will contribute to more effective treatment decision-making for patients with serious diseases such as malignant cancers.

## 5 Conclusion

In this paper, we present a one-stop research platform SenseCare that provides a large set of AI algorithms for medical image segmentation, registration, detection and other tasks that can help clinicians and radiologists conduct various clinic-oriented translational research programs, such as lung cancer diagnosis and surgical planning, efficient pathological image analysis, pelvic tumor and limb surgical planning, coronary artery disease diagnosis and modeling, etc. In addition to the built-in AI algorithms, SenseCare also provides several tools for users to develop and deploy customized AI models efficiently. The AI toolkits and other appearing functional modules such as advanced visualization, web-based access and multi-center deployment in SenseCare can efficiently boost clinical research programs and applications towards smart healthcare.

## Data Availability

The raw data supporting the conclusions of this article will be made available by the authors, without undue reservation.
